# Behavior of FDG-avid supradiaphragmatic lymph nodes in PET/CT throughout primary therapy in advanced serous epithelial ovarian cancer: a prospective study

**DOI:** 10.1186/s40644-019-0215-7

**Published:** 2019-05-29

**Authors:** Maren Laasik, Jukka Kemppainen, Annika Auranen, Sakari Hietanen, Seija Grénman, Marko Seppänen, Johanna Hynninen

**Affiliations:** 1 Department of Obstetrics and Gynecology, Turku University Hospital, University of Turku, Kiinamyllynkatu 4-8, 20521 Turku, Finland; 2 Department of Nuclear Medicine, Turku PET Center, Turku University Hospital, University of Turku, Kiinamyllynkatu 4-8, 20521 Turku, Finland; 3 Department of Obstetrics and Gynecology, Tampere University Hospital, University of Tampere, Teiskontie 35, 33521 Tampere, Finland

**Keywords:** FDG-PET/CT, Ovarian cancer, FDG-avid supradiaphragmatic lymph nodes

## Abstract

**Background:**

Epithelial ovarian cancer (EOC) typically spreads intra-abdominally, but preoperative evaluation with FDG PET/CT often reveals metabolically active supradiaphragmatic lymph nodes (sdLNs). Their clinical significance and behavior during treatment has not been established.

**Methods:**

EOC patients with PET positive sdLNs at diagnosis were prospectively followed with PET/CT after primary chemotherapy and at the first recurrence. In each patient, 2 most active LNs in 5 different supradiaphramatic regions were evaluated and the size and changes in FDG uptake (SUVmax) were recorded. The patients´ overall response to primary treatment was defined with RECIST criteria. The behavior of sdLNs during chemotherapy were compared in treatment responders and non-responders. Recurrence patterns were monitored.

**Results:**

Forty-one patients with 127 PET/CT scans were systematically evaluated. In pretreatment scan, 76% (31/41) of patients had FDG-avid sdLNs in multiple anatomical sites. Only a minority (22/136) of the sdLNs were enlarged in size, but their histopathologic confirmation by biopsy was not possible. Only 6/41 patients had FDG-avid sdLNs in a single surgically approachable site. The sdLNs became inactive during primary chemotherapy more often in the RECIST responders compared to the non-responders (HR 1.46 (95%CI: 1.09–1.96), *p* = 0.002). The size and SUVmax values did not predict treatment outcome. In 50% of the responders the same sdLNs reactivated when recurrence occurred. Persistent post-treatment metabolic activity did not predict earlier disease relapse (*p* = 0.59).

**Conclusion:**

The behavior of metabolically active sdLNs during chemotherapy supports their metastatic nature. Due to their distribution to multiple regions, the benefit of removal of reachable sdLNS seems unlikely.

**Trial registration:**

NCT, NCT01276574. Registered 1 September 2010.

**Electronic supplementary material:**

The online version of this article (10.1186/s40644-019-0215-7) contains supplementary material, which is available to authorized users.

## Introduction

The vast majority of EOC is diagnosed at an advanced stage [1] and optimal removal of intraabdominal tumor bulk forms a major prognostic factor for survival [2, 3]. The need to extend cytoreductive surgery outside the abdominal cavity has recently been a focus of interest.

Increasing evidence indicates that abnormal [^18^F]-fluoro-2-deoxy-D-glucose (FDG) accumulation in sdLNs is a common finding in advanced EOC [4–6]. International Federation of Gynecology and Obstetrics (FIGO) staging system requires histopathological verification of extra-abdominal metastases [7]. Pretreatment positron emission computed tomography (PET/CT) may reveal small supradiaphragmatic lymph node metastases (sdLNM) unreachable for sampling. In addition, the common presence of FDG-avid sdLNs suggests that many FIGO stage IIIC patients actually have extra-abdominal disease. Radiological suspicion of sdLNM can cause confusion while staging the OC and choosing the treatment modality.

Although there are limited data on the clinical significance of the radiologically detected extra-abdominal disease spread, it has been suggested that FDG-avid sdLNs may be a predictive parameter in advanced EOC for the failure of optimal cytoreduction [8], the probability of neoadjuvant chemotherapy (NACT) as the primary intervention [9], the lower rate of complete initial treatment response [8, 9] and inferior survival [10]. The standard surgical management of EOC is aimed at the removal of the intra-abdominal lesions.

We have earlier presented results from a prospectively recruited cohort of advanced EOC patients, where 20/30 patients were found to have PET positive sdLNs at the time of diagnosis [4]. In the present study, we aim to evaluate the FDG-avid sdLNs’ pretreatment characteristics, and their response to first line chemotherapy and patterns of recurrence by means of a thorough radiologic follow-up in patients without intrathroracic debulking. In addition, we aim to assess the predictive value of sdLNs size and SUVmax to treatment outcome, and the impact of sdLNs metabolic response to progression free survival (PFS).

## Methods

### Patients

The current patient cohort was collected as part of prospective clinical MUPET trial (ClinicalTrials.gov Identifier: NCT01276574) and treated at Turku University Hospital’s Department of Obstetrics and Gynecology between Oct 2009 and Feb 2014.

**Fig. 1:**
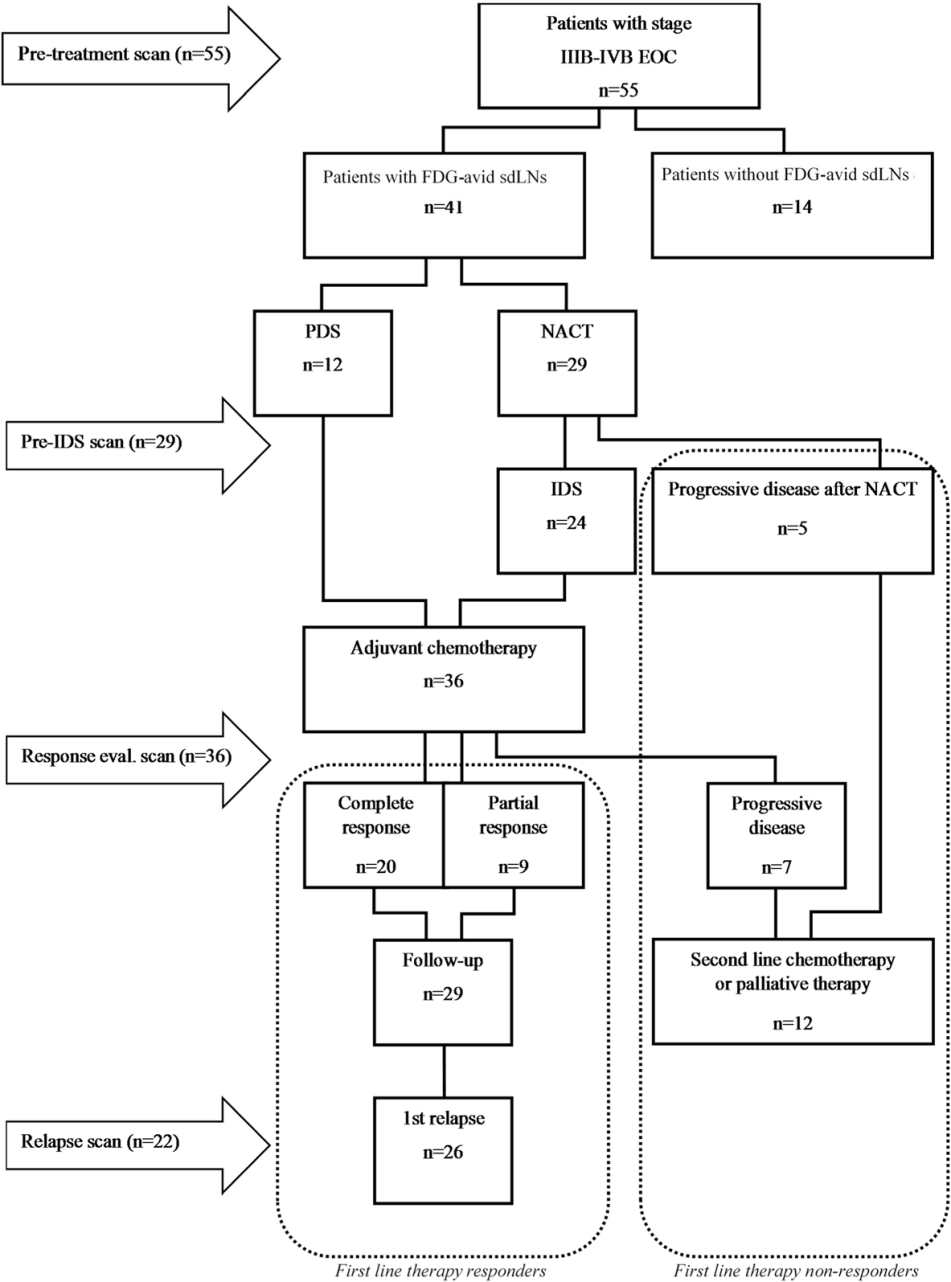
Study outline and overview of the treatment modalities

Fifty-five patients with stage IIIB-IVB serous EOC were included, 41 (74%) of them had FDG-avid sdLNs in the pretreatment FDG-PET/CT scan and comprised the final study cohort. The patients with other previous malignancies and diabetes were excluded. The patients’ characteristics are presented in Table 1. FDG-PET/CT scans were performed at the following stages: a) the preoperative assessment, b) after NACT prior to interval debulking surgery (IDS), c) after the first line standard platinum-taxane based chemotherapy, and d) at the time of the first relapse of the disease (Fig. 1). The number of FDG-PET/CT scans was 2–4 per patient.

**Table 1:** Patients’ characteristics

Variables	Patients (n)	%
Total	41	100
Age, median (years,range)	63 (30–80)	
*FIGO stage*		
IIIB	2	4.9
IIIC	18	43.9
IVA	6	14.6
IVB	15	36.6
*Histology*		
High grade serous	37	90.2
Low grade serous	4	9.8
*Treatment strategy*		
Primary debulking surgery	12	29.3
Neoadjuvant chemotherapy	29	70.7
Interval debulking surgery	24	58.5
*PDS outcome*		
No residual tumor	3	25.0
Residual tumor size < 1 cm	4	33.3
Residual tumor size > 1 cm	5	41.7
*IDS outcome*		
No residual tumor	6	25.0
Residual tumor size < 1 cm	17	70.8
Residual tumor size > 1 cm	1	4.2
*Primary treatment outcome*		
Complete response	20	48.8
Partial response	9	22.0
Stable disease	0	0
Progressive disease	12	29.2
*Recurrence rate after completion of first line therapy (N = 29)*		
All patients	26	89.7
Complete response	14	46.2
Partial response	12	53.8

SdLNs were not surgically removed in any of the patients. The selection of the treatment schedule was based on clinical examination, preoperative FDG-PET/CT, and diagnostic laparoscopy or laparotomy. Ultrasound guided biopsies from FDG-PET/CT positive sdLNs (*N* = 5) were taken when feasible. Patients who underwent surgery (*N* = 36) received 3–6 cycles of adjuvant chemotherapy after operation and patients with progressive disease after NACT (*N* = 5) were not operated and were changed over second line chemotherapy.

### PET/CT scanning procedure and data analysis

A whole-body contrast-enhanced FDG-PET/CT was performed with either a 64-row Discovery STE or a VCT (General Electric Medical Systems, Milwaukee, WI, USA). Imaging studies were performed prior to the treatment, after NACT, 4 weeks after the last cycle of the adjuvant chemotherapy and at the first relapse of the disease. All patients fasted for a minimum of 6 h and their serum glucose level was controlled before the intravenous injection of the 4 Mbq/kg ^18^F-FDG isotope. The low-dose PET/CT (kV 120, Smart mA range 10–80) from skull base to mid-thigh was performed 50–60 min after the tracer injection. It was followed by a whole-body diagnostic high dose contrast-enhanced CT scan (kV 120, Smart mA range100–440), after the automated intravenous injection of a contrast agent. PET images were reconstructed with a 128 × 128 matrix size in a fully 3D mode using an ML-OSEM reconstruction algorithm. Imaging analysis was performed using an ADW4.5 workstation.

Two dedicated nuclear medicine experts analyzed the integrated FDG-PET/high dose contrast-enhanced CT images. PET imaging analysis was performed using an ADW 4.5 workstation. The evaluation was systematic and included all anatomical sites. PET positive (FDG-avid) findings were collected into a detailed worksheet in order to compare the change in the standardized uptake value (SUVmax) and size in the PET/CT scans of the same LNs, taken at different time points. Typical physiologically active FDG-avid sites were excluded. The SUVmax values were corrected for body weight and injected dose.

For LN based analysis, supradiaphragmatic area was divided into five anatomical regions: cardiophrenic, parasternal, mediastinal (including hilar area), axillary and subclavian [4]. The two most metabolically active LNs from each anatomical site were evaluated. The SUVmax values were calculated, but no specific cutoff value for SUVmax defining the LNs as metastatic was applied. In patients receiving NACT FDG-avid retroperitoneal lymph node metastases in paraaortic and parailiacal sites were evaluated.

The patients’ response to first line treatment was evaluated according to the criteria of Response Evaluation Criteria in Solid Tumors (RECIST) version 1.1 [11] and CA-125 criteria of The Gynecological Cancer Intergroup (GCIC) [12]. The treatment response evaluation was based on contrast enhanced CT and the serum marker CA-125, not PET/CT. Metabolic response in an individual sdLN was considered complete when the SUVmax value did not differ from that of the surrounding background after initial therapy, and partial when the reduction of FDG uptake was a minimum of 30% [13]. All of the patients had regular check-ups with clinical status and serum CA-125 controls after completion of first line chemotherapy. Imaging studies were performed when clinical symptoms suggesting relapse occurred. Recurrence of the disease was defined as constantly elevated levels of CA-125 tumor marker or anatomical progression in CT scan.

In order to compare the behavior of the FDG-avid sdLNs in patients with different clinical outcomes, the patients who according to RECIST 1.1 and GCIC criteria had obtained at least a partial response after the completion of adjuvant chemotherapy were considered first line therapy responders (responders), while those with a stable or progressive disease where categorized as non-responders.

### Statistical analyses

All statistical analyses were performed using JMP Pro 12 software from SAS. Continuous variables between the two groups were compared using a Mann-Whitney U-test. Fisher’s exact test was used in the analysis of contingency tables to summarize the relationship between categorical variables. Kaplan-Meier survival curves for PFS of study patients were compared with a log rank test for all variables. PFS was defined as the time interval from the diagnosis until the disease progression or death. *P*-values < 0.05 were considered statistically significant.

## Results

In total, 127 PET/CT scans were analyzed. Forty-one patients (75%) of 55 had FDG-avid sdLNs (*N* = 136) in preoperative FDG-PET/CT. The responders group consisted of 29 patients with 97 FDG-avid sdLNs prior to therapy and the non-responders group included 12 patients with 39 FDG-avid sdLNs. One to nine sdLNs per patient (mean 3.3) were analyzed.

### FDG-avid sdLNs in preoperative PET/CT

**Fig. 2:**
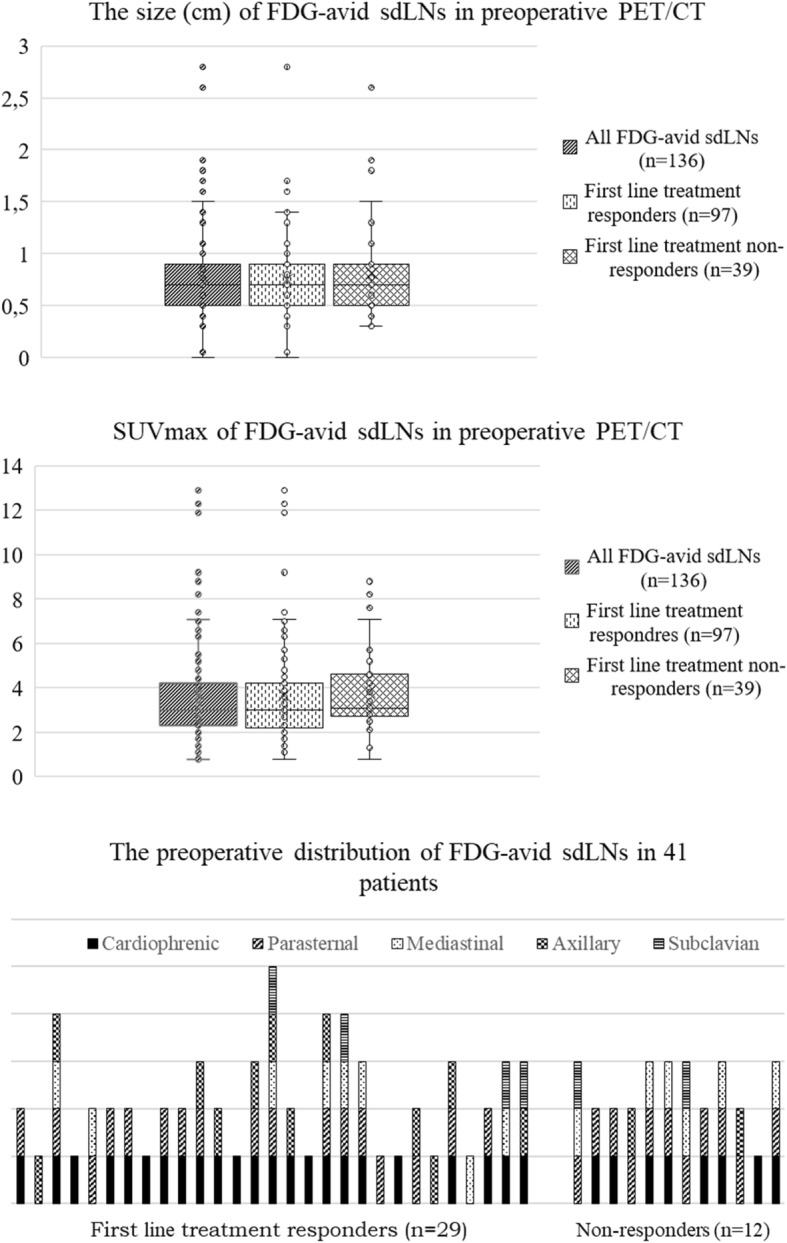
The size and the SUVmax of FDG-avid supradiaphragmatic lymph nodes (sdLNs) in the preoperative PET/CT of 41 patients with advanced EOC

Of the 136 FDG-avid sdLNs, 16% (22/136) sdLNs were enlarged (short axis ≥ 10 mm). The size, SUVmax values and distribution of FDG-avid sdLNs in preoperative PET/CT is presented in Fig. 2. There was no statistically significant difference in the average size and SUVmax values of FDG-avid sdLNs in preoperative imaging between the responders and non-responders (*p* = 0.84 and 0.29).

Of the 41 patients, 31 (76%) had metabolically active sdLNs in multiple anatomical sites (Table 2, Fig. 2). 76% (31/41) had preoperatively FDG-avid LNs in the cardiophrenic area, that could potentially be resected in surgery. Notably, in 6 patients this was the only FDG-avid sdLN station.

**Table 2:** Distribution and characteristics of FDG-avid supradiaphragmatic lymph nodes (sdLNs) in PET/CT

	Pre-operative PET/CT			Treatment response evaluation PET/CT			The 1st relapse PET/CT		
The anatomical site of FDG-avid sdLNs	Patients with FDG-avid sdLNs N (%)	Mean short axis mm(±sd)	Mean SUVmax (±sd)	Patients with FDG-avid sdLNs N (%)	Mean short axis mm(±sd)	Mean SUVmax (±sd)	Patients with FDG-avid sdLNs N (%)	Mean short axis mm(±sd)	Mean SUVmax (±sd)
*Responders group (N = 29)*									
Parasternal	17 (59)	5.6(±2.3)	2.7(±0.8)	2 (7)	2.2(±1.9)	1.7(±0.5)	7 (32)	5.7(±2.5)	4.0(±2.3)
Subclavian	4 (14)	8.0(±2.3)	4.8(±1.9)	1 (3)	8.0(±3.2)	4.8	2 (9)	8.0(±3.6)	6.4(±4.3)
Axillary	12 (41)	9.1(±4.4)	4.4(±3.9)	5 (17)	6.3(±3.2)	2.0(±0.8)	4 (18)	14.9(±7.0)	11.1(±9.4)
Mediastinal	8 (28)	8.5(±2.5)	4.3(±1.8)	3 (10)	5.5(±2.9)	2.9(±0.3)	9 (41)	7.3(±1.4)	5.6(±3.5)
Cardiophrenic	23 (79)	7.7(±4.4)	3.4(±2.1)	3 (10)	4.8(±1.6)	1.9(±0.05)	7 (32)	7.8(±6.2)	3.8(±3.4)
*Total*	*29 (100)*	*7.6(±3.8)*	*3.6(±2.4)*	*10 (34)*	*5.5(±2.9)*	*2.3(±0.9)*	*13 (59) of 22*	*8.6(±5.5)*	*6.0(±5.6)*
*Multiple sites*	*20 (69)*			*4 (14)*			*8 (36)*		
*Single site*	*9 (31)*			*6 (20)*			*5 (23)*		
*Non-responders group (N = 12)*									
Parasternal	11 (92)	4.9(±1.4)	2.9(±0.5)	5 (42)	8.5(±8.7)	1.8(±0.8)			
Subclavian	2 (17)	14.3(±5.3)	6.2(±0.9)	1 (8)	5.0	3.8(±0.7)			
Axillary	2 (17)	13.0(±5.1)	5.7(±3.1)	0	–	–			
Mediastinal	6 (50)	12.4(±6.7)	5.2(±2.4)	3 (25)	8.2(±2.2)	3.3(±1.6)			
Cardiophrenic	8 (67)	6.8(±1.6)	3.2(±1.2)	6 (50)	5.1(±1.4)	2.8(±1.2)			
*Total*	*12 (100)*	*8.1(±5.1)*	*3.8(±2.0)*	*7 (58)*	*6.8(±4.9)*	*2.9(±1.4)*			
*Multiple sites*	*11 (92)*			*3 (25)*					
*Single site*	*1 (8)*			*4 (33)*					

Distribution and characteristics of FDG-avid supradiaphragmatic lymph nodes (sdLNs) in PET/CT in patients with advanced stage epithelial ovarian cancer at different time points of the disease

### The behavior of FDG-avid sdLNs in response to the primary treatment

The behavior of sdLNs during primary therapy reflected the patients’ overall chemotherapy response.

The metabolic response of 97 preoperatively detected FDG-avid sdLNs in responders and 39 in non-responders group were evaluated. After the completion of the first line chemotherapy, 96% (93/97) of the FDG-avid sdLNs in responders group responded metabolically to the treatment, 82.5% (80/97) with complete and 13.4% (13/17) partial metabolic response. In the non-responders group, despite the disease progression elsewhere, 22 (56%) of the preoperatively FDG-avid 39 sdLNs showed complete metabolic response, whereas 21% (8/39) showed partial metabolic response.

The sdLNs in the responders group more frequently showed a complete metabolic resolution after primary treatment compared to the non-responders group, hazard ratio 1.46 (95%CI: 1.09–1.96) (*p* = 0.002).

Of the sdLNs that were still metabolically active in the response evaluation PET/CT, there had been a mean decline in the SUV max values of 42% in the responders group compared to only 20% in the non-responders group (*p* = 0.02).

The same tendency, although not statistically significant, was detected when analyzed at the level of the individual patients: the 35% (10/29) of patients in the responders group and 71% (7/12) in the non-responders group had FDG avid sdLNs in the response evaluation scan (*p* = 0.18) (Table 2).

Similar to retroperitoneal LNM, the metabolic response to chemotherapy in FDG-avid sdLNs was already detectable after NACT (Table 3).

**Table 3:** The metabolic response of supradiaphragmatic and retroperitoneal lymphnodes to neoadjuvant chemotherapy

	Response to first line treatment	Supradiaphragmatic lymphnodes	Retroperitoneal lymphnodes	*p value*
Complete response after NACT	responders	65% (39/60)	68% (23/34)	*0.82*
	non-responders	30% (11/37)	40% (8/20)	*0.55*
Mean decrease in SUVmax in sdLNs with partial metabolic response	responders	42%	67%	*0.13*
	non-responders	20%	27%	*0.90*

### The FDG-avid sdLNs profile at the first disease relapse

**Fig. 3:**
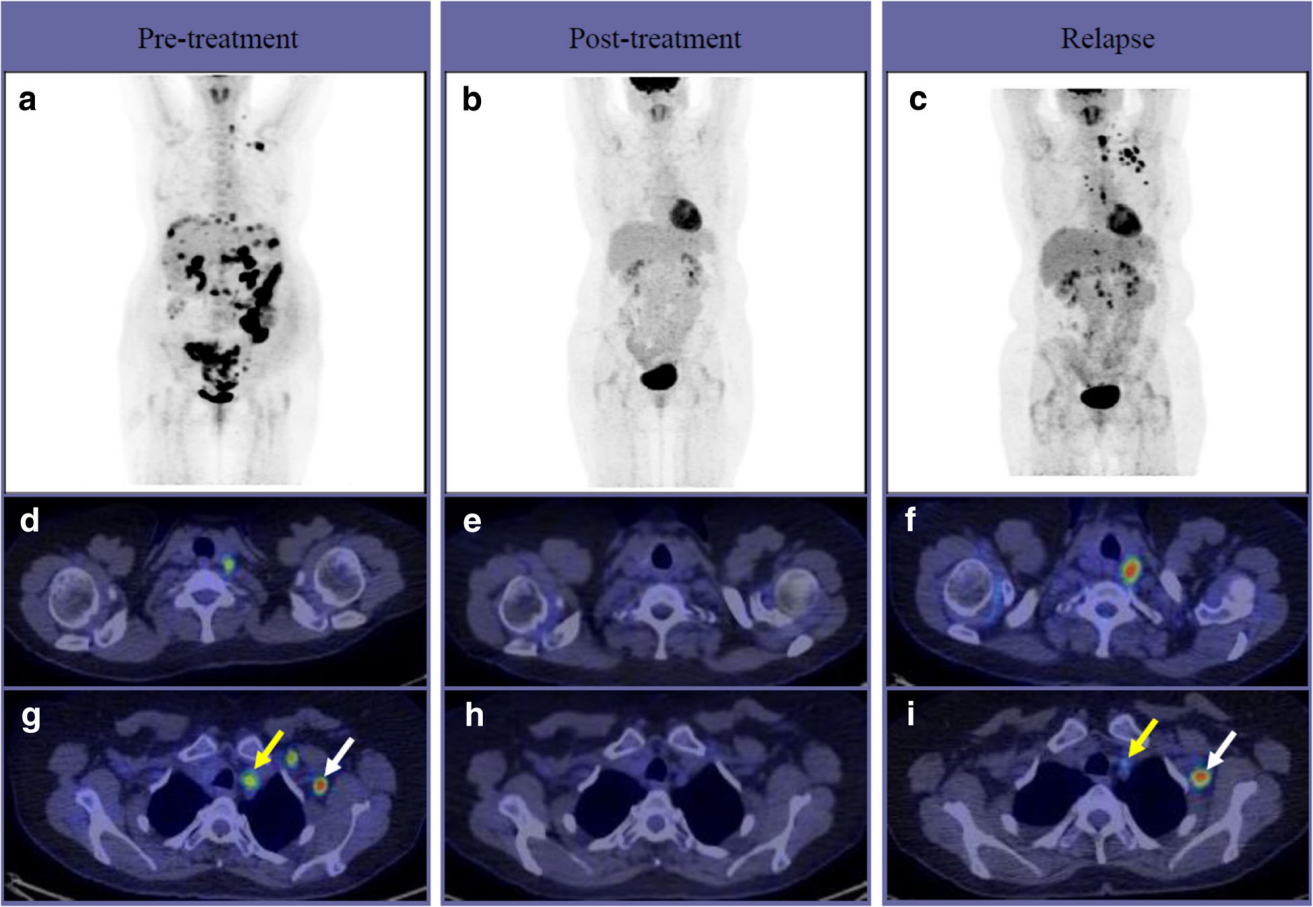
The preoperative whole body PET/CT maximum intensity projection view of the patient with FIGO stage IIIC high grade EOC show, in addition to the abdominal involvement, an extensive FDG uptake in cardiophrenic, parasternal, mediastinal, subclavicular, neck and axillary lymph nodes (**a**). After 3 cycles of NACT, an interval debulking surgery (with no gross residual disease) and 3 cycles of standard adjuvant taxane-platinum based chemotherapy the FDG uptake has been normalized (**b**). PET/CT performed 11 months after the end of primary treatment shows metabolic reactivation of paraaortal and supradiaphragmatic lymph nodes as well as new metabolically active supradiaphragmatic lymph nodes (**c**). Transaxial PET/CT fusion images show the preoperatively detected FDG-avid lymph node profile during treatment and relapse in the neck (d,e,f) as well as in the axillary (white arrow) and mediastinal (yellow arrow) (g,h,i) regions

The FDG-avid sdLNs which responded to first line chemotherapy often reactivated during disease recurrence. Disease recurrence in the thorax alone was rare. Figure 3 presents the behavior of preoperatively detected FDG-avid sdLNs of our study patient in FDG-PET/CT during primary treatment until the first recurrence.

The responders (*n* = 29) had regular follow-up visits after completion of primary therapy. Within the median follow-up time of 35.8 months (95% Cl: 31.7–41.0), 90% (26/29) responders experienced disease recurrence and 69% (20/29) died. The median PFS of responders was 14 months (95% Cl: 1.1–18.4). There was no difference in median PFS of patients with complete and partial metabolic response in FDG-avid sdLNs (13,6 vs 14.9 months), respectively (*p* = 0.59).

The overall distribution of the disease when relapse occurred is presented in Additional file 1: Table S1. In 50% (11/22) of first line responders the same sdLNs activated when recurrence was detected.

Biopsies from FDG-avid sdLNs were pre-operatively taken from five patients and malignant histology was confirmed in all 5 cases (4 axillary and 1 subclavicular LNM). Two of the confirmed sdLN metastases were enlarged (≥10 mm) and 3 normal (< 10 mm) in size. The mean preoperative SUV max of the confirmed sdLN metastases was 3.3 and their metabolic response to first line chemotherapy was complete. The size and preoperative SUVmax values of the confirmed sdLN metastases did not differ statistically that from the not biopsied FDG-avid sdLNs.

## Discussion

Detection of metabolically active sdLNs have been reported in a number of studies using PET/CT imaging [5, 6, 9, 10, 14, 15]. These findings often lack histologic confirmation, since anatomical sites such as the parasternal and mediastinal lymph chain are unreachable for biopsy. Radiologic follow-up is an indirect method to confirm their malignant nature. To the best of our knowledge, the current study is the first one designed for prospective and systematic monitoring of the behavior of FDG-avid sdLNs with repeated PET/CT scans during treatment and relapse.

Our data suggest that the FDG-avid sdLNs do represent metastatic infiltration and are not artefactual or reactive changes. In line with previous studies [5, 10, 14], our follow up including 127 meticulously analyzed PET/CT scans showed that the vast majority of sdLNs accumulating FDG are normal in size. The metastatic nature of FDG avid sdLNs is suggested by two findings. Firstly, similar to the patients’ other lesions (including retroperitoneal LNM), FDG-avid sdLNs respond to first line chemotherapy both in a per-lesion and in a per-patient assessment. The metabolic response was similar in FDG-avid sdLNs and retroperitoneal LNM already after NACT prior any debulking surgery was performed.

Secondly, in half of the patients, the same sdLNs reactivated and enlarged during the recurrence. The number of histopathologically confirmed sdLNM was small (*N* = 5). However, FDG-PET/CT finding led to histological sampling of the hot spot and upstaging the disease from FIGO stage IIIC to IVB in all of these patients. The confirmed sdLNM also showed complete metabolic response to first line chemotherapy and often reactivated at the time of recurrence. In addition to decrease in SUVmax values, sdLNs that were detectable after first line treatment decrease in size after treatment and thickened when relapse was detected. However, the change was only a few millimeters and sdLNs were all along normal in size.

The prognostic significance of the PET positive sdLNs in pretreatment scan may be limited. Neither the size nor the SUVmax of sdLNs in the pretreatment scan predicted the patients’ primary therapy outcome. Compared to patients with complete metabolic resolution in sdLNs post treatment, partial metabolic response to the initial therapy was not associated with earlier disease relapse. In addition, in the majority of sdLNs in non-responders group showed some metabolic response, albeit progression of the disease in the abdominal cavity. This weakens the prognostic value of sdLNs as their metabolic response to the treatment does not seem to reflect the disease status in the abdomen nor instructs the possible further treatment.

In agreement with previous reports [10, 16, 17], we found that among patients with suspected extra-abdominal disease the recurrence to the thorax alone is rare and the most common site of the first relapse is the abdomen. The multidirectional migration of malignant cells and the reseeding of the primary tumor by metastasis has been demonstrated in human prostate cancer [18]. Post treatment residual metastatic infiltration in sdLNs may theoretically also represent a reservoir of malignant cells in OC. In present study, some patients had large volume residual disease in abdominal cavity after surgery and therefore any conclusions on role of sdLNs as cancer reservoir cannot be concluded.

Resection of enlarged cardiophrenic LNs has been reported to be a safe and feasible procedure for patients with advanced EOC [19–22]. However, there is controversial data about the survival benefit of extending PDS outside the abdominal cavity [10, 22, 23] and no consensus over the cutoff for pathologic cardiophrenic LNs. Values ranging between 5 and 10 mm have been suggested [19, 24, 25]. In our cohort, the majority of FDG-avid sdLNs were normal in size (when cutoff ≥10 mm for enlarged was used) and they were most commonly localized in the cardiophrenic and parasternal areas. However, only a small proportion of patients had all of the metabolically suspicious sdLNs in surgically approachable area.

The role of routine retroperitoneal lymphadenectomy of normal size nodes has been questioned in advanced EOC, since it is reported not to improve patients’ outcome [26, 27]. It can be anticipated that this is also liable to be the case with sdLNs. Garbi et al. [21] recently reported no recurrences in the cardiophrenic angle when debulked during PDS. Since they did not use PET/CT preoperatively or in the follow up, the status of other sdLNs was not known. In a recent study of Lee et al [10], primary debulking of sdLNM did not improve survival. In our study, only a minority of advanced EOC patients with intrathoracic disease had FDG-avid sdLNs in a single surgically approachable anatomical site. That may raise questions about the benefit of removing only the suspicious cardiophrenic LNs. We suggest that the centers committing cardiophrenic LNs resection should consider performing FDG-PET/CT covering also thorax area prior to surgery and during the follow-up in order to clarify the clinical significance of detected FDG-avid sdLNs and the survival benefit of cardiophrenic surgery in EOC.

This study has certain limitations including the absence of histological verification of sdLNMs in the majority of patients (36/41), restriction in the assessment of prognostic significance due to the small number and heterogenous characteristics of patients. The strengths of our study are the prospectively designed, systematic and detailed evaluation of the FDG-avid sdLNs behavior with PET/CT throughout the disease, and the long follow-up time of the patients.

## Conclusions

FDG-avid sdLNs are common in patients with advanced EOC. FDG-avid sdLNs are often unsuspicious in conventional imaging modalities, and unreachable for histopathological verification. In addition, they are often distributed over multiple anatomical sites which precludes complete surgical removal. Our comprehensive follow-up study supports the metastatic nature of FDG-avid sdLNs detected with PET/CT. Unremoved FDG-avid sdLNs responded metabolically to chemotherapy and often reactivated during disease recurrence. The prognostic significance of favorable metabolic treatment response in detected FDG-avid sdLNs in EOC patients is limited. For survival analyses further controlled studies with an adequate control group are needed.

## Additional file

Additional file 1: **Table S1.** The distribution of metastases at the time of recurrence among first line therapy responders (*N* = 22). (DOCX 32 kb)

## Abbreviations

EOC: Epithelial ovarian cancer
FDG-PET/CT: [^18^F]-fluoro-2-deoxy-D-glucose positron emission computed tomography
FIGO: The International Federation of Gynecology and Obstetrics
GCIC: The Gynecological Cancer Intergroup
IDS: Interval debulking surgery
LNM: Lymph node metastases
NACT: Neoadjuvant chemotherapy
PDS: Primary debulking surgery
PFS: Progression free survival
RECIST: Response Evaluation Criteria in Solid Tumors
sdLNMs: Supradiaphragmatic lymph node metastases
sdLNs: Supradiaphragmatic lymph nodes
SUVmax: Standardized uptake value

## References

[1] DeSantis CE, Lin CC, Mariotto AB (2014). Cancer treatment and survivorship statistics, 2014. CA Cancer J Clin.

[2] Chang SJ, Hodeib M, Chang J (2013). Survival impact of complete cytoreduction to no gross residual disease for advanced-stage ovarian cancer: a meta-analysis. Gynecol Oncol [Internet] Elsevier Inc.

[3] Bristow R, Tomacruz R, Armstrong DK (2002). Survival effect of maximal cytoreductive surgery for advanced ovarian carcinoma during the paltinum era: a meta-analysis. J Clin Oncol.

[4] Hynninen J, Auranen A, Carpén O, et al. FDG PET/CT in staging of advanced epithelial ovarian cancer: frequency of supradiaphragmatic lymph node metastasis challenges the traditional pattern of disease spread. Gynecol Oncol. 2012. 10.1016/j.ygyno.2012.04.023 22542580

[5] Fruscio R, Sina F, Dolci C, et al. Preoperative 18F-FDG PET/CT in the management of advanced epithelial ovarian cancer. Gynecol Oncol. 2013. 10.1016/j.ygyno.2013.09.024 24076062

[6] Nam EJ, Yun MJ, Oh YT, et al. Diagnosis and staging of primary ovarian cancer: correlation between PET/CT, Doppler US, and CT or MRI. Gynecol Oncol. 2010. 10.1016/j.ygyno.2009.10.059 19926121

[7] Prat J (2014). Staging classification for cancer of the ovary, fallopian tube, and peritoneum. Int J Gynaecol Obstet United States.

[8] Bats AS, Hugonnet F, Huchon C (2012). Prognostic significance of mediastinal 18F-FDG uptake in PET/CT in advanced ovarian cancer. Eur J Nucl Med Mol Imaging.

[9] Raban O, Peled Y, Krissi H, et al. The significance of paracardiac lymph-node enlargement in patients with newly diagnosed stage IIIC ovarian cancer. Gynecol Oncol. 2015. 10.1016/j.ygyno.2015.05.007 26001327

[10] Lee IO, Lee J-Y, Kim HJ, et al. Prognostic significance of supradiaphragmatic lymph node metastasis detected by 18F-FDG PET/CT in advanced epithelial ovarian cancer. BMC Cancer [Internet] BioMed Central; 2018 [cited 2019];18:1165. Available from: https://bmccancer.biomedcentral.com/articles/10.1186/s12885-018-5067-1 10.1186/s12885-018-5067-1 PMC6260780 30477469

[11] Eisenhauer EA, Therasse P, Bogaerts J (2009). New response evaluation criteria in solid tumours: revised RECIST guideline (version 1.1). Eur J Cancer England.

[12] Rustin Gordon John Sampson, Vergote Ignace, Eisenhauer Elizabeth, Pujade-Lauraine Eric, Quinn Michael, Thigpen Tate, du Bois Andreas, Kristensen Gunnar, Jakobsen Anders, Sagae Satoru, Greven Kathryn, Parmar Mahesh, Friedlander Michael, Cervantes Andres, Vermorken Jan (2011). Definitions for Response and Progression in Ovarian Cancer Clinical Trials Incorporating RECIST 1.1 and CA 125 Agreed by the Gynecological Cancer Intergroup (GCIG). International Journal of Gynecologic Cancer.

[13] Wahl R. L., Jacene H., Kasamon Y., Lodge M. A. (2009). From RECIST to PERCIST: Evolving Considerations for PET Response Criteria in Solid Tumors. Journal of Nuclear Medicine.

[14] Im Hyung-Jun, Kim Yong-il, Paeng Jin Chul, Chung June-Key, Kang Soon-Beom, Lee Dong Soo (2011). Retrocrural Lymph Node Metastasis Disclosed by 18F-FDG PET/CT: A Predictor of Supra-diaphragmatic Spread in Ovarian Cancer. Nuclear Medicine and Molecular Imaging.

[15] Signorelli M, Guerra L, Pirovano C, et al. Detection of nodal metastases by 18F-FDG PET/CT in apparent early stage ovarian cancer: a prospective study. Gynecol Oncol. 2013. 10.1016/j.ygyno.2013.08.022 23988414

[16] Jamieson A, Sykes P, Eva L, et al. Subtypes of stage IV ovarian cancer; response to treatment and patterns of disease recurrence. Gynecol Oncol [Internet]. Elsevier Inc.; 2017; Available from: http://linkinghub.elsevier.com/retrieve/pii/S009082581730879X 10.1016/j.ygyno.2017.05.023 28549816

[17] Perri T, Ben-Baruch G, Kalfon S (2013). Abdominopelvic cytoreduction rates and recurrence sites in stage IV ovarian cancer: is there a case for thoracic cytoreduction. Gynecol Oncol [Internet]. Elsevier Inc..

[18] Hong MKH, Macintyre G, Wedge DC, et al. Tracking the origins and drivers of subclonal metastatic expansion in prostate cancer. Nat Commun [internet]. Nat Publ Group. 2015;6:6605 Available from: https://www.ncbi.nlm.nih.gov/pmc/articles/PMC4396364/10.1038/ncomms7605 PMC4396364 25827447

[19] Prader S, Harter P, Grimm C, et al. Surgical management of cardiophrenic lymph nodes in patients with advanced ovarian cancer. Gynecol Oncol. 2016. 10.1016/j.ygyno.2016.03.012 26972337

[20] Cowan Renee A., Tseng Jill, Murthy Vijayashree, Srivastava Radhika, Long Roche Kara C., Zivanovic Oliver, Gardner Ginger J., Chi Dennis S., Park Bernard J., Sonoda Yukio (2017). Feasibility, safety and clinical outcomes of cardiophrenic lymph node resection in advanced ovarian cancer. Gynecologic Oncology.

[21] Garbi A, Zanagnolo V, Colombo N (2017). Feasibility of Transabdominal Cardiophrenic Lymphnode Dissection in Advanced Ovarian Cancer Initial Experience at a Tertiary Center.

[22] Nasser S, Kyrgiou M, Krell J (2017). A review of thoracic and mediastinal Cytoreductive techniques in advanced ovarian Cancer: extending the boundaries. Ann Surg Oncol United States.

[23] Mert I, Kumar A, Sheedy SP, et al. Clinical significance of enlarged cardiophrenic lymph nodes in advanced ovarian cancer: Implications for survival. Gynecol Oncol [Internet]. Elsevier Inc. 2017; Available from: http://linkinghub.elsevier.com/retrieve/pii/S0090825817314464. 10.1016/j.ygyno.2017.10.024 29129390

[24] Forstner R, Kinkel K, Spencer JA (2010). ESUR guidelines : ovarian cancer staging and follow-up. Eur J Radiol.

[25] Kim Tae-Hyung, Lim Myong Cheol, Kim Se Ik, Seo Sang-Soo, Kim Sun Ho, Park Sang-Yoon (2015). Preoperative Prediction of Cardiophrenic Lymph Node Metastasis in Advanced Ovarian Cancer Using Computed Tomography. Annals of Surgical Oncology.

[26] Bois A, Reuss A, Harter P (2010). Potential Role of Lymphadenectomy in Advanced Ovarian Cancer : A Combined Exploratory Analysis of Three Prospectively Randomized Phase III Multicenter Trials.

[27] Harter Philipp, Sehouli Jalid, Lorusso Domenica, Reuss Alexander, Vergote Ignace, Marth Christian, Kim Jae-Weon, Raspagliesi Francesco, Lampe Björn, Aletti Giovanni, Meier Werner, Cibula David, Mustea Alexander, Mahner Sven, Runnebaum Ingo B., Schmalfeldt Barbara, Burges Alexander, Kimmig Rainer, Scambia Giovanni, Greggi Stefano, Hilpert Felix, Hasenburg Annette, Hillemanns Peter, Giorda Giorgio, von Leffern Ingo, Schade-Brittinger Carmen, Wagner Uwe, du Bois Andreas (2019). A Randomized Trial of Lymphadenectomy in Patients with Advanced Ovarian Neoplasms. New England Journal of Medicine.

