# Supplementary material for: Behavior of FDG-avid supradiaphragmatic lymph nodes in PET/CT throughout primary therapy in advanced serous epithelial ovarian cancer: a prospective study
Source: Cancer Imaging. 2019 May 29;19:27. doi: 10.1186/s40644-019-0215-7 (PMC6542004; doi:10.1186/s40644-019-0215-7)
Supplement: Supplementary file 1 — Table S1. The distribution of metastases at the time of recurrence among first line therapy responders (N = 22). (DOCX 32 kb) [file 40644_2019_215_MOESM1_ESM.docx]

**Additional file 1: Table S1** The distribution of metastases at the time of recurrence among first line therapy responders (N=22)

| **Location of metastases** | **Patients, n (%)** | | |
| --- | --- | --- | --- |
| FDG-avid suprdiaphragmatic lymphnodes (sdLNs) | Total | In single site | In multiple site |
| Total | 13 (59%) | 5 (23%) | 8 (36%) |
| Parasternal | 7 | 1 | 6 |
| Subclavicular | 2 | 0 | 2 |
| Mediastinal | 9 | 2 | 7 |
| Axillary | 4 | 1 | 3 |
| Cardiophrenic | 7 | 1 | 6 |
| Retroperitoneal lymph node metastases | 14 (64%) | | |
| Intraabdominal metastases | 17 (77%) | | |
| Other distant metastases | 1 (4%), *lungs* | | |
| Ascites | 7 (32%) | | |
|  |  | | |
